# DNA methylation in APOE: The relationship with Alzheimer's and with cardiovascular health

**DOI:** 10.1002/trc2.12026

**Published:** 2020-04-27

**Authors:** Jure Mur, Daniel L. McCartney, Rosie M. Walker, Archie Campbell, Mairead L. Bermingham, Stewart W. Morris, David J. Porteous, Andrew M. McIntosh, Ian J. Deary, Kathryn L. Evans, Riccardo E. Marioni

**Affiliations:** ^1^ Department of Psychology University of Edinburgh Edinburgh UK; ^2^ Centre for Genomic and Experimental Medicine Institute of Genetics and Molecular Medicine University of Edinburgh Edinburgh UK; ^3^ Division of Psychiatry Centre for Clinical Brain Sciences University of Edinburgh Edinburgh UK

**Keywords:** Alzheimer's disease, APOE, biomarker, cardiovascular disease, cholesterol, DNA methylation

## Abstract

**Introduction:**

Genetic variation in the apolipoprotein E (*APOE*) gene is associated with Alzheimer's disease (AD) and risk factors for cardiovascular disease (CVD). DNA methylationat *APOE* has been associated with altered cognition and AD. It is unclear if epigenetic marks could be used for predicting future disease.

**Methods:**

We assessed blood‐based DNA methylation at 13 CpGs in the *APOE* gene in 5828 participants from the Generation Scotland (GS) cohort. Using linear mixed models regression, we examined the relationships among *APOE* methylation, cognition, cholesterol, the family history of AD and the risk for CVD.

**Results:**

DNA methylation at two CpGs was associated with the ratio of total cholesterol and HDL cholesterol, but not with cognition, family history of AD, or the risk of CVD.

**Discussion:**

*APOE* methylation is associated with the levels of blood cholesterol, but there is no evidence for the utility of *APOE* methylation as a biomarker for predicting AD or CVD.

## 1 INTRODUCTION

### 1.1 The APOE gene

The apolipoprotein E (*APOE)* gene is located on chromosome 19, with two single nucleotide polymorphisms (SNPs) in its fourth exon defining three alleles, ε2, ε3, and ε4, resulting in the production of three isoforms of the apoE protein, apoE2, E3, and E4.^1^ The *APOE* ε4 genotype is a well‐known risk factor for Alzheimer's disease (AD)^2^, ^3^ and a prominent candidate in cardiovascular research.^4^, ^5^ The protein is expressed in various tissues, including the brain.^6^ It acts as a ligand for members of the low‐density lipoprotein (LDL) receptor family and is involved in the clearance of lipoproteins and cholesterol from the circulatory system.^7^, ^8^

*APOE* exhibits an allele‐specific association with risk of AD. Possession of the ε4‐allele confers an increased risk, while the ε2‐allele is protective.^2^ The isoforms of apoE differ in their binding affinity for lipoproteins and LDL receptors, and differentially influence levels of serum cholesterol.^9^ The Ε2 variant reduces the levels of total and LDL cholesterol, while the E4 variant raises them.^6^ However, the connection between *APOE* and cardiovascular disease (CVD) is less robust. While there is some evidence for an increased risk of CVD‐related death in carriers of the ε4‐allele,^1^ this has not always been observed.^10^

### 1.2 APOE methylation in AD and CVD

Epigenetic modifications have been associated with many human disorders.^11^ DNA methylation (DNAm) is most commonly observed as the addition of a methyl group to the 5‐position of cytosine in the context of a cytosine‐guanine dinucleotide (CpG^12^). Many studies have found a link between AD and DNAm.^12^, ^13^ Candidate gene studies for *APOE* have shown that DNAm in this gene is associated with dementia^14^, ^15^, ^16^, ^17^ with neuritic amyloid plaque burden,^18^ and with cognitive ability.^19^

Associations have been suggested between epigenetic mechanisms and risk factors for cardiovascular disease.^20^, ^21^, ^22^, ^23^ A recent systematic review identified 34 candidate gene studies on DNAm in CVD.^4^ However, few studies have explored modifications specifically in *APOE*, and there have been some conflicting results: while Karlsson et al.^15^ found no evidence for differences in *APOE* DNAm in blood between patients with CVD and healthy controls, Ji et al.^24^ reported *APOE* hypermethylation in the blood of patients with coronary heart disease compared to controls. No AD‐ or CVD‐related associations were reported in the EWAS catalogue^25^; for cg14123992, cg04406254, cg05501958, and cg21879725, associations with other traits were reported (Table S1 in supporting information).

To help clarify this role of *APOE*, we use data from Generation Scotland (GS), a large population‐based cohort, the size of which is several times greater than samples in previous studies, providing a robust analysis of *APOE* DNAm and cognitive and vascular health. We included both AD and CVD in our analysis for the following reasons: First, *APOE* is both highly relevant to the genetics of AD, as well as of importance to cardiovascular health due to its function of clearing lipoproteins and cholesterol from the blood.^7^, ^8^ Second, the state of current knowledge is relatively limited for *APOE* DNAm in AD and for *APOE* DNAm in CVD. Third, CVD and cardiovascular risk factors strongly influence the risk of AD and are a relevant research goal in the context of AD.

We characterize blood‐based DNAm in the promoter region, 2nd and 3rd exons and introns, and 4th exon of the *APOE* gene, and explore associations among *APOE* DNAm and a variety of markers of cognitive function, AD, vascular health, and CVD. Due to the importance of developing new clinical biomarkers for health outcomes before the onset of disease (the neuropathological hallmarks of dementia start to appear decades prior to onset), we restrict our sample to ages between 30 and 65 years.

**RESEARCH IN CONTEXT**

1. Based on a review of the primary literature, and on recent reviews and meta‐analyses, associations have been reported between DNAm in the apolipoprotein E (*APOE*) gene and both cognitive function and cardiovascular health. However, these have been from smaller studies (n ≤ 1076). Here, we further explore these associations in a very large cohort with 5828 participants.
2. Our findings do not support the utility of *APOE* DNAm in blood as a biomarker to predict the future risks of Alzheimer's disease (AD) and cardiovascular disease. However, the findings suggest a potential association between *APOE* DNAm and the levels of blood cholesterol.
3. Longitudinal research on changes in DNAm is required to elucidate the exact timing of epigenetic changes as they relate to cognition and AD. Additionally, replication and mechanistic studies on the observed association between *APOE* DNAm and blood cholesterol are needed.

## 2 METHODS

### 2.1 The sample

GS^26^, ^27^ is a family‐based cohort of more than 22,000 individuals from Scotland (aged 18 to 99 years) that were genotyped and extensively phenotyped during the baseline assessment between the years 2006 and 2011. Blood‐based genome‐wide DNAmwere generated in 9551 individuals as part of the substudy Stratifying Resilience and Depression Longitudinally (STRADL^28^). All participants provided informed consent.

### 2.2 DNA Methylation and APOE measurements

DNAm in peripheral blood samples was profiled in two analysis sets (Set 1: n = 5087 in 2017 and Set 2: n = 4450 in 2019), using the Illumina HumanMethylationEPIC BeadChip as described previously.^29^ Briefly, low quality samples, probes with low detection rates, and participants for which the predicted sex did not match the recorded sex were excluded (see Methods S1 in supporting information).

We removed related participants from Set 1 using a genetically determined relationship cut‐off of >0.05 (GCTA GREML^30^) to reduce the potential influence of shared genetics on the findings. The participants in Set 2 were unrelated to each other and to those in Set 1. We restricted the analysis to CpGs located on chromosome 19 between 45,409,039 and 45,412,650 bp, which corresponded to the region encompassing the *APOE* gene (UCSC GRCh37/hg19 genome build). To restrict the age of our sample, we removed participants younger than 30 years and older than 65 years. After combining the two sets, our final sample consisted of a total of 13 CpGs in 5828 participants (Figure S1 in supporting information).

*APOE* haplotype status was determined using Taqman technology at the Clinical Research Facility, Western General Hospital, Edinburgh. Based on the identity of the nucleotides at SNP positions rs429358 and rs7412, participants with the ε3/ε3 haplotype were classified as ε3 carriers, participants with the ε2/ε2 and ε2/ε3 haplotypes were classified as ε2 carriers, and participants with the ε3/ε4 and ε4/ε4 haplotypes were classified as ε4 carriers. The 126 participants (2.2%) with the ε2/ε4 genotype were not included in analyses in which *APOE* carrier status was implemented as a variable.

### 2.3 Characterization of APOE methylation

**FIGURE 1:**
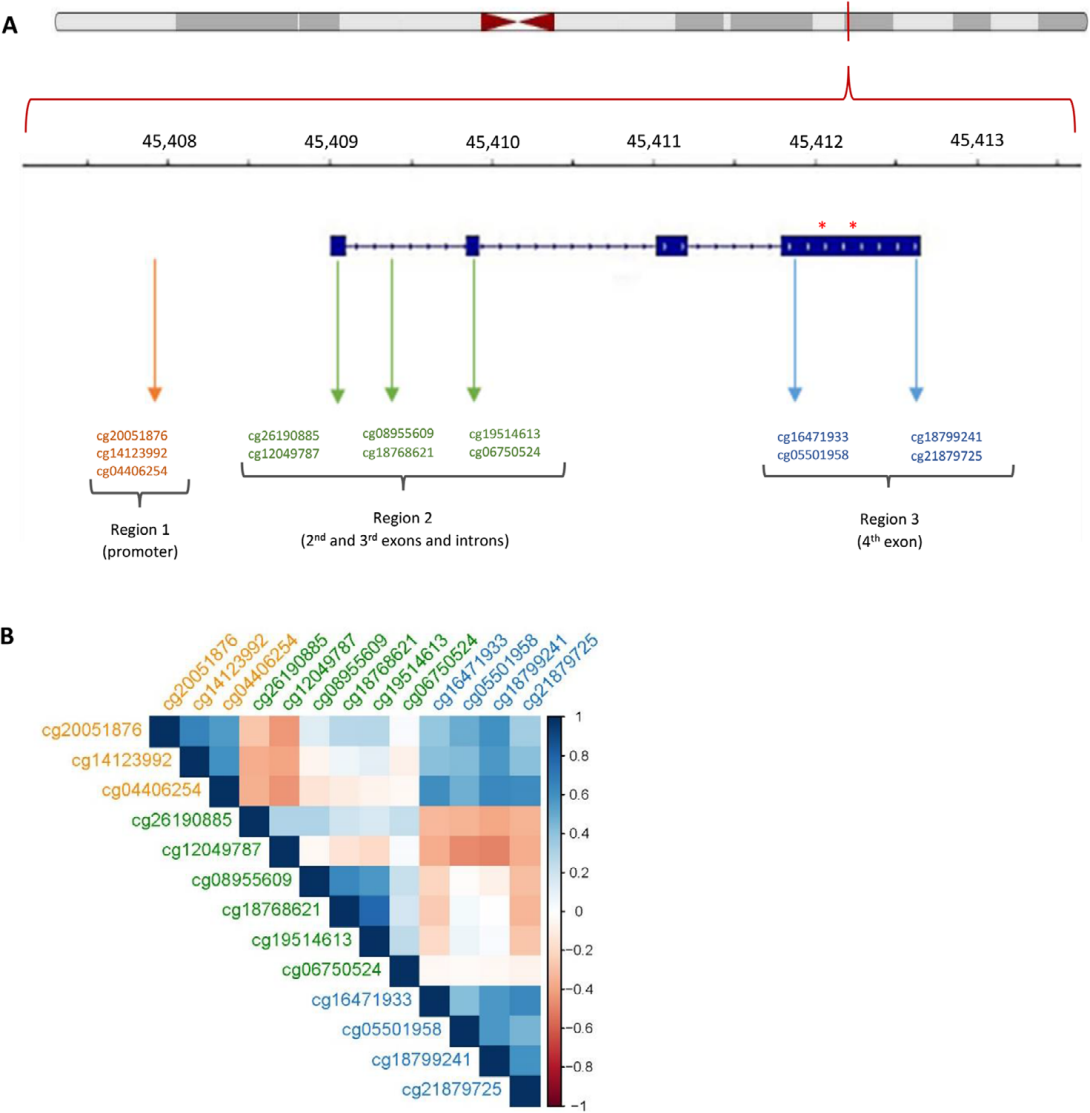
Structure of the apolipoprotein E (*APOE*) locus and the location of CpGs (**A**), and correlations among CpGs in the *APOE* gene (**B**). **A**: The top panel shows the location of APOE on the chromosome, the second panel indicates the location on *APOE* in kb, the third panel shows the structure of *APOE*, with exon regions thickened; the asterisks denote the locations of SNPs rs429358 and rs 7412 that confer the *APOE* genotype

We examined the DNAm status of 13 CpGs in the *APOE* gene (Figure 1A). Twelve CpGs exhibited similar DNAm levels to those previously described (Table S2^15^, ^16^, ^22^ in supporting information), whereas one of them (cg20051876, which is unique to the Illumina EPIC array) had not been reported before. Based on the Illumina‐annotated locations of the CpGs on the chromosome, their relative distances to each other, and their DNAm levels, the CpGs were classified into three groups: hypermethylated (each site >50% mean DNAm) and lying in the promoter region (region 1: cg20051876, cg14123992, cg04406254), hypomethylated (each site <50% mean DNAm) and lying in the region encompassing the first two exons and introns (region 2: cg26190885, cg12049787, cg08955609, cg18768621, cg19514613, cg06750524), and hypermethylated and lying in the 4th exon (region 3: cg16471933, cg05501958, cg18799241, cg21879725).

### 2.4 Cognitive variables

Four measures of cognitive function^27^ were evaluated: (1) the Wechsler digit symbol test as a measure of the speed of information processing, (2) the letter‐based (C, F, L) phonemic verbal fluency test as a measure of executive function, (3) the Mill Hill Vocabulary test (combined junior and senior synonyms) as a measure of crystallized ability, and (4) the Wechsler immediate and delayed Logical Memory tests (one story) as a measure of verbal declarative memory. Participants with a zero value were judged as resulting from non‐participation and were recorded as missing. A measure of global cognitive function (cognitive‐g) was derived by applying principal component analysis (PCA) to the four cognitive measures, with cognitive‐g defined as the first unrotated principal component. This factor explained 44% of the total variance from all four cognitive measures, with loading values ranging between 0.47 and 0.53.

### 2.5 Approximations for the risk of AD and of CVD

When completing the questionnaires, participants had reported on the presence of “AD” and “heart disease” for themselves and for their family members. Only three participants reported having AD, and 203 participants (3.5%) reported having CVD. We therefore created approximate measures of risk that were based on reports by participants about these illnesses for close relatives (parents, siblings, and grandparents). For both AD and CVD, each relative was assigned either 0 or 1 (0: absence of the disorder, 1: presence of the disorder) based on the report of the participant. To derive each risk measure, we calculated the weighted sums of family records for each participant as follows:

$$\begin{array}{ccc} \mathrm{risk} & = & 0.5\;\times (\mathrm{mother}+\mathrm{father}+\mathrm{sister}+\mathrm{brother})+\;0.25\\ & & \times \;(\mathrm{either}\;\;\mathrm{grandparent}) \end{array}$$

The choice of weights used for a given family member was based on kinship/relatedness between that relative and the participant. The participants that self‐reported as having AD or CVD were excluded from the models that included the risk of AD or the risk of CVD as predictor variables, respectively. Due to a low number of relatives with AD and a consequential skew in the distribution of AD risk, AD risk was transformed into a categorical variable AD class (low risk: no close relatives with AD, n = 4854; high risk: at least one close relative with AD, n = 974). Using this approach, we were able to estimate the risk for each disorder for every participant in the study.

### 2.6 Covariates

For alcohol consumption, we did not include participants that have quit drinking (n = 373) or whose answers relating to recent alcohol consumption according to their own assessment did not correspond to their usual drinking pattern (n = 1557). Information on smoking was processed as described previously^31^; we did not include participants that have quit smoking (n = 673). The sample sizes for the fully adjusted models (see below) thus depended on the included covariates with missing values. Smoking was assessed in pack years and alcohol consumption in units per week. Activity levels were recorded in minutes per week; two different versions of the questionnaire were used in the study, with each participant completing only one of them. We therefore combined the two versions so that the responses by all participants were on the same scale (see Methods S2 in supporting information).

### 2.7 Statistical analysis

For all numerical variables, outliers—defined as scores beyond four interquartile ranges from the median—were excluded. For the analyses exploring effects on DNAm as an outcome, linear mixed models were used; for analyses exploring effects on AD class as an outcome, logistic‐regression models were used.

Each model was run twice: First as a basic adjusted model that included the variable(s) as defined in the hypothesis as predictors, and cell counts, processing batch, and analysis set as covariates. Next, a fully adjusted model was run that included additional covariates (sex, smoking, alcohol consumption, education, deprivation index, and—in some models—physical activity, heart rate, body mass index [BMI], *APOE* genotype; see Methods S3 in supporting information).

The predictor‐specific covariates used in the fully adjusted model varied according to the predictor variable of the model and, whenever possible, corresponded with previous studies.^15^, ^22^ Both the basic and the fully adjusted model included as covariates the batch in which the samples of a given participant were processed on the array (fitted as a random effect on the intercept) and the estimated proportions of CD8+ and CD4 + T‐cells, natural killer cells, B cells, monocytes, and granulocytes. Cell composition correction controls for the fact that DNAm patterns can be confounded by the heterogeneity of cells in the tissue used for analysis.^32^

The threshold for statistical significance was Bonferroni‐corrected for each CpG. All continuous variables (except the variable *age* when used as a predictor) were transformed to have a mean of 0 and a standard deviation of 1. When the predictor was a categorical variable, analysis of variance was performed, comparing the model of interest with the same model excluding the predictor variable.

All statistical analyses were performed in R.

## 3 RESULTS

### 3.1 Sample characteristics

Among the 5828 participants, 3399 (58.3%) were female and 2429 (41.7%) were male. The age range was 30 to 65 years and the mean age was 52.7 years (Table 1). *APOE* genotype frequencies were comparable to those described previously for the British population (Table 1).^10^, ^33^DNAm measures resembled previous reports^19^ and most CpGs appeared normally distributed upon visual inspection (Figure S2 in supporting information). Correlations between DNAm levels in blood (this study) and brain tissue (publicly available datasets) for the 13 CpGs in this study ranged from –0.30 to 0.51 for the whole brain (IMAGE‐CpG: https://han-lab.org/methylation/default/imageCpG,^34^ last accessed October 13, 2019), from –0.26 to 0.40 for Brodmann area 20 (BECon: https://redgar598.shinyapps.io/BECon/,^35^ last accessed October 13, 2019), and from –0.21 to 0.20 for the entorhinal cortex (https://epigenetics.essex.ac.uk/bloodbrain/,^36^ last accessed October 13, 2019), with most CpGs exhibiting low correlations between brain and blood DNAm (Table S2).

**TABLE 1:** Descriptive statistics and frequencies of genotypes and alleles/carriers in the sample

Trait	Mean^a^	SD^b^
Age	53.0^a^	14.0^a^
Years in education^c^	4.0^a^	3.0^a^
Deprivation quintile^d^	4.0^a^	3.0^a^
Processing speed (Wechsler digit symbol test)	71.2	15.6
Executive function (letter‐based verbal fluency test)	41.1	11.7
Verbal ability (Mill Hill Vocabulary test)	30.1	4.5
Verbal declarative memory (Wechsler logical memory test immediate and delayed)	30.9	7.7
Heart rate (beats/min)	63.7	10.3
Ratio/quotient of total cholesterol and HDL cholesterol	3.7^a^	1.6^a^
Alcohol units per week	8.0^a^	12.0^a^

Trait	N	%
Sex (female)	3399	58.3
AD in close relatives	974	16.7
CVD in close relatives	3474	59.6

Genotype/carrier	n	%
ε2/ε2	31	0.5
ε2/ε3	647	11.4
ε2/ε4	126	2.2
ε3/ε3	3352	59.0
ε3/ε4	1370	24.1
ε4/ε4	156	2.7
ε2‐carrier	678	12.2
ε3‐carrier	3352	60.3
ε4‐carrier	1526	27.5

^a^The median is given when appropriate.

^b^The interquartile range is given when appropriate.

^c^A 10‐grade classification‐system, where 0: 0 years, 1: 1–4 years, 2: 5–9 years, 3: 10–11 years, 4: 12–13 years, 5: 14–15 years, 6: 16–17 years, 7: 18–19 years, 8: 20–21 years, 9: 22–23 years, 10: ≥24 years.

^d^Scottish Index of Multiple Deprivation (SIMD), 2011.

Abbreviations: AD, Alzheimer's disease; CVD, cardiovascular disease

### 3.2 Associations between CpGs

The correlations in DNAm for all pairwise combinations between the 13 CpGs ranged from –0.49 to 0.78, with a mean absolute correlation of 0.32 (Figure 1B, Table S3 in supporting information). After correcting for multiple comparisons, 68/78 correlations were statistically significant at *P *< 6.4 × 10^‐4^ (= 0.05/78), with 38 (60%) positive, and 30 (40%) negative. CpGs within a region tended to be methylated to a similar extent: all significant correlations among CpGs within region 1 (3/3, 100%) and within region 3 (6/6, 100%), and most of the significant correlations among CpGs within region 2 (11/13, 85%) were positive. Moreover, there was a tendency for CpGs in regions 2 and 3 to be methylated in opposite directions: most of the significant correlations (17/22, 77%) were negative. In contrast, regions 1 and 3 tended to be methylated in the same direction, with all significant correlations (12/12, 100%) positive. Most CpGs exhibited strong associations with imputed cell proportions (estimates range: ‐0.371 to 0.345, with 50/65 associations significant at <7.6 × 10^‐4^; Table S4 in supporting information).

### 3.3 Association between DNA methylation and genotype

**FIGURE 2:**
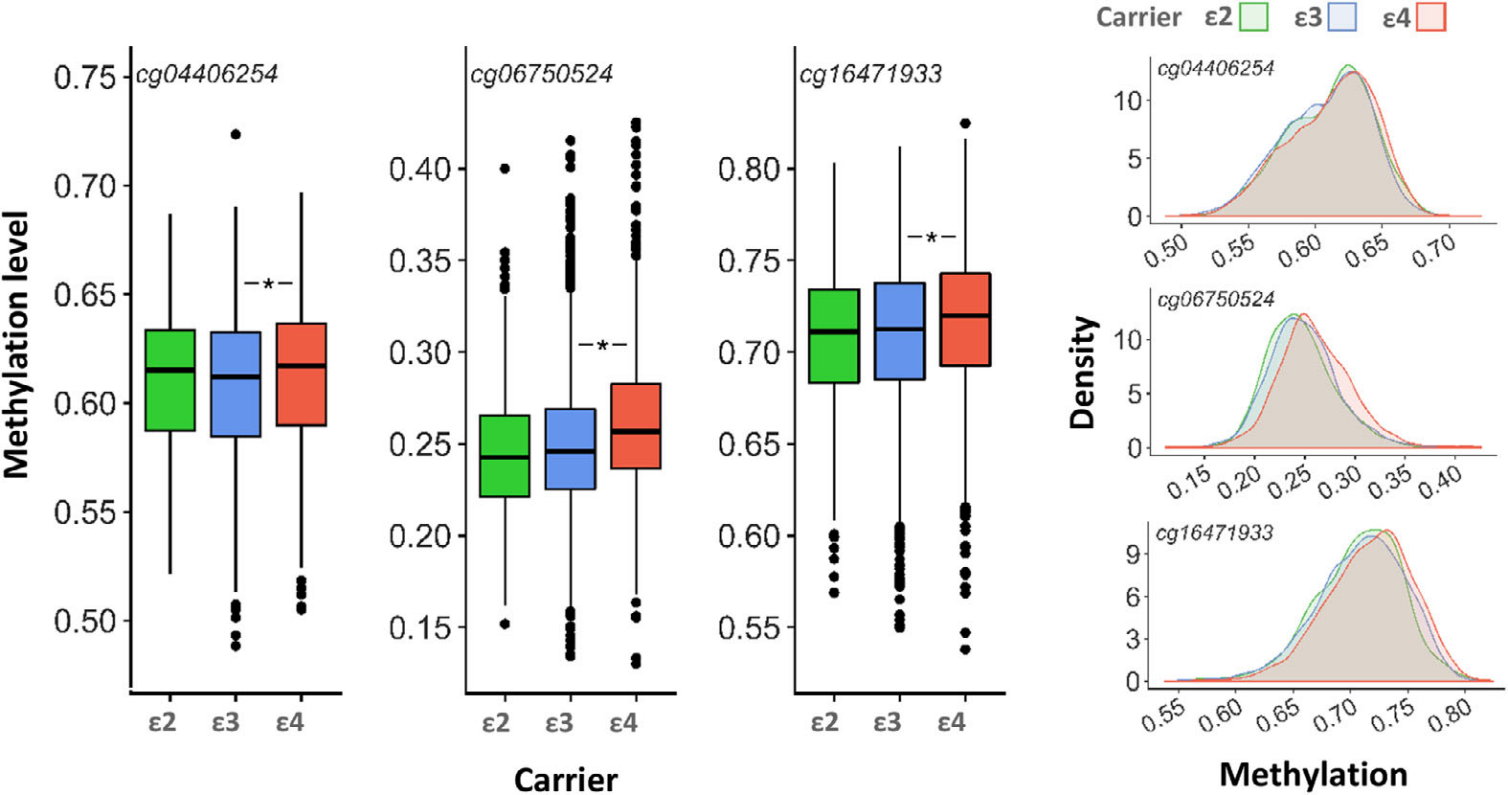
Boxplots (left) and density plots (right) for differences in DNAm levels between groups with different apolipoprotein E (*APOE*) carrier status

After correcting for multiple testing, there was evidence for an association between *APOE* carrier status and DNAm at five CpGs in the fully adjusted models: cg14123992 (η^2 ^= 0.003, *P* = 3.9 × 10^‐4^), cg04406254 (η^2 ^= 0.004 *P* = 4.0 × 10^‐6^), cg06750524 (η^2 ^= 0.022, *P* = 8.6 × 10^‐16^), cg16471933 (η^2 ^= 0.014, *p* = 2.4 × 10^‐16^), and cg21879725 (η^2 ^= 0.003, *P* = 1.3 × 10^‐4^; Figure 2, Table S5 in supporting information). Among these five CpGs, the CpGs from regions 1 (cg14123992, cg04406254) correlated relatively strongly with one another (r = 0.61) and with CpGs in region 3 (cg16471933, cg21879725; r = 0.42‐0.61). The CpG from region 2 (cg06750524) exhibited weak correlations with the other four CpGs (–0.07‐[–0.04]). This suggests only two or three independently associated CpGs with *APOE* carrier status. Post‐hoc testing between the *APOE* groups showed that at all five CpGs the associations were due to higher DNAm levels in ε4 carriers compared to ε3 carriers.

### 3.4 Age‐dependent drift in DNA methylation

**FIGURE 3:**
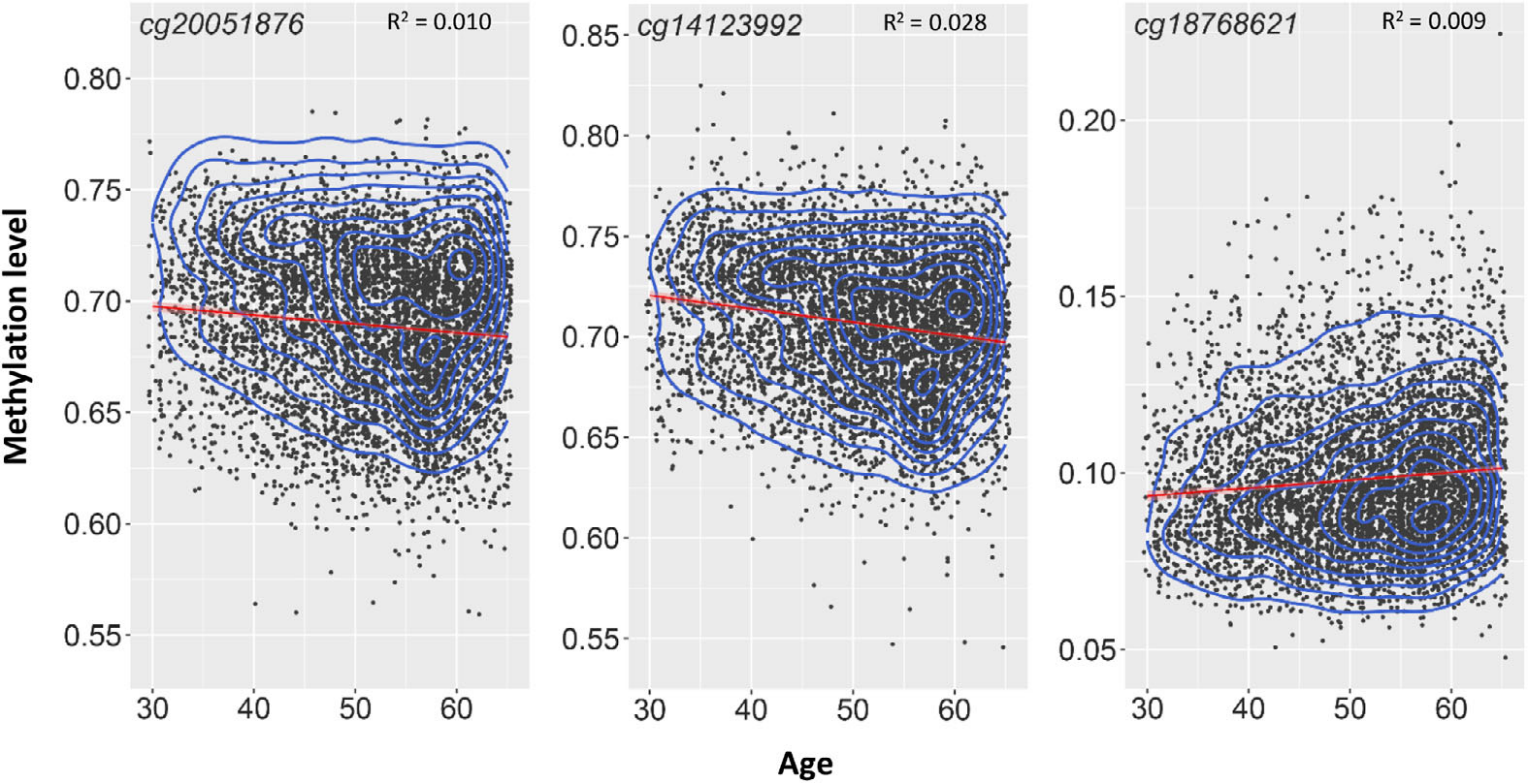
Relationship between age (years) and DNAm (beta‐levels)

In the basic‐adjusted model, 7/13 CpGs showed an association with age after correction for multiple testing (R^2^ range: 4.0 × 10^‐5^–0.03; Table S6 in supporting information). Three CpGs remained significant after correction for multiple testing in the fully adjusted model (Figure 3, Table S6). Most CpGs within a region exhibited the same direction of change with respect to aging. CpGs in the generally hypomethylated region 2 tended to have greater DNAm with older age, whereas CpGs in the generally hypermethylated regions 1 and 3 tended to have lower DNAm with older age. None of the CpGs in our analyses were in a list of age‐related variable methylated positions (aVMPs^37^). However, the Breusch‐Pagan test for heteroscedasticity indicated non‐random variation in the residuals by age for all CpGs in both the basic and the fully adjusted models (Table S7 in supporting information).

### 3.5 APOE methylation and cognitive function

We observed no association between general cognitive ability and DNAm in the fully adjusted regression models after correction for multiple testing (Table S8 in supporting information). There were associations between the individual cognitive tests and DNAm levels in the basic but not fully adjusted regression models (Table S9 in supporting information).

### 3.6 APOE methylation and the risk of AD

To determine whether *APOE* carrier status was associated with AD class (0: absence of the disorder, 1: presence of the disorder), a logistic regression was run, with AD class as the outcome variable and *APOE* carrier status as the predictor. The Wald Chi‐squared test confirmed a general effect of *APOE* carrier status on AD class in the basic adjusted model (*χ^2^*=43.1, *P*=4.3 × 10^‐10^) and in the fully adjusted model (*χ^2^*=32.7, *P*=1.0 × 10^‐7^). The ε4 allele was significantly associated with the high‐risk AD class both in the basic (OR=1.66, *P*=1.5 × 10^‐10^) and fully adjusted models (OR=1.73, *P*=2.3 × 10^‐7^). We observed no associations between DNAm levels and AD risk in the basic or fully adjusted models after correcting for multiple testing (Table S10 in supporting information).

### 3.7 APOE methylation and the risk of CVD

**FIGURE 4:**
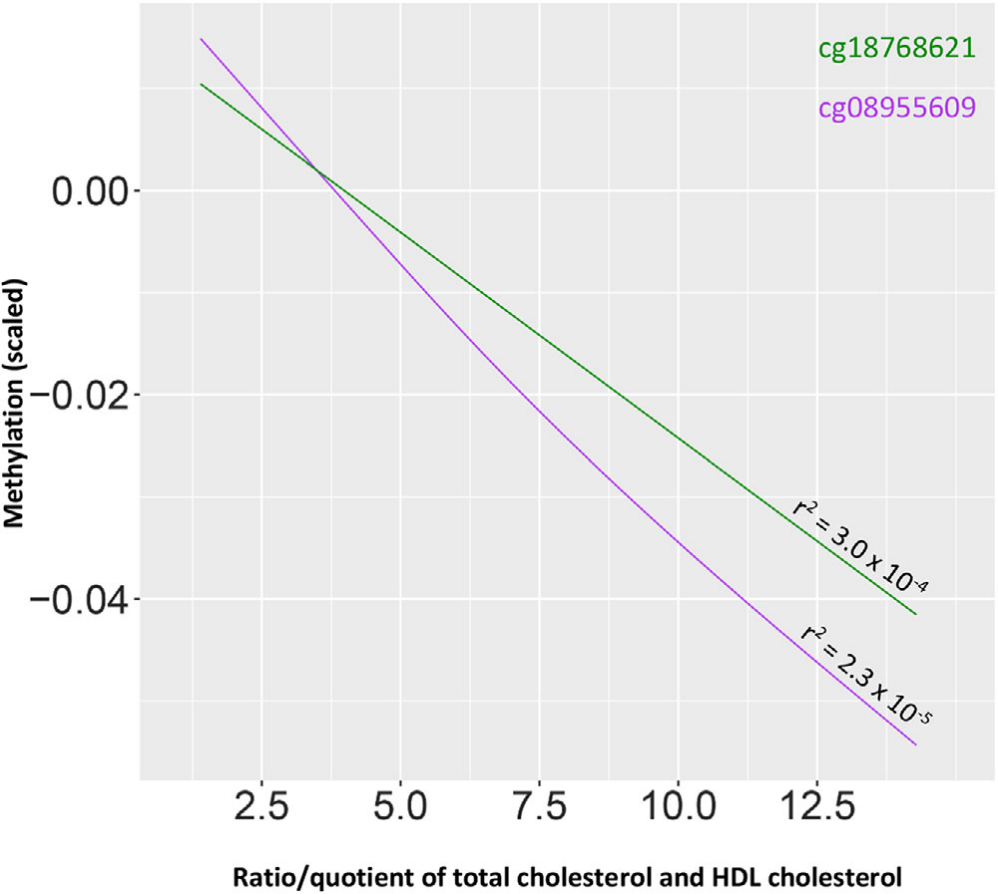
Relationship between ratio/quotient of total cholesterol and high‐density lipoprotein cholesterol, and DNAm

We observed no association between *APOE* methylation and the risk measure of CVD (Table S11 in supporting information), nor was there any relationship between *APOE* genotype and the risk measure of CVD. However, *APOE* genotype was associated with the ratio/quotient of total cholesterol and high‐density lipoprotein (HDL) cholesterol in the fully adjusted model (*χ^2^*=68.5, *P*=1.3 × 10^‐15^). Specifically, both the ε2 and ε4 carriers differed in the ratio/quotient of total cholesterol and HDL cholesterol compared to ε3 carriers in the fully adjusted model (ε2: estimate=‐0.22, SE=0.048, *P*=6.4 × 10^‐6^; ε4: estimate=0.21, SE=0.036, *P*=1.2 × 10^‐8^). The ratio of ratio/quotient of total cholesterol and HDL cholesterol was significantly associated with DNAm levels after correcting for multiple testing at three CpGs in the fully adjusted models: cg08955609, cg18768621, and cg16471933 (Table S12 in supporting information). Due to the strong association between *APOE* genotype and cholesterol levels, the fully adjusted model was rerun with *APOE* genotype as covariate; cg08955609 (estimate=−0.065, SE=0.019, *P*=6.7 × 10^‐4^) and cg18768621 (estimate=−0.059, SE=0.017, *P*=4.3 × 10^‐4^) remained statistically significant (Figure4, Table S12). The validity of CVD risk as a risk measure for CVD was confirmed by running a logistic regression model, with self‐reported CVD as an outcome measure and CVD risk as a predictor variable, with covariates as in the fully adjusted model above (OR=1.81, *P*=2.2 × 10^‐10^).

## 4 DISCUSSION

### 4.1 Correlations between CpGs and age‐drift in DNA methylation

In this study, we used DNAm and phenotypic data from a large cohort, GS, to explore DNAm in the *APOE* gene and its association with risk factors for AD, CVD, and blood cholesterol.

We observed correlations among CpGs, which had been reported before.^19^ Compared with Liu et al.,^19^ the correlations observed in our study were stronger and more of them were negative. In contrast to the findings of Karlsson et al.,^15^ the DNAm levels of five CpGs correlated with *APOE* carrier status for the different *APOE* alleles. This difference may be due to the increased power of our study over that of Karlsson et al.^15^

We observe associations between most CpGs and imputed proportions of white blood cells. It has been suggested that relative numbers of cell subtypes might be caused by the same regulatory perturbations that give rise to certain phenotypes.^38^ We did not explore potential associations between AD or cardiovascular risk factors, and cell proportions in this study, but it represents an interesting possibility for future research.

We replicated a finding by Ma et al.^22^ of age drift in DNAm in the *APOE* gene: 3/13 CpGs showed age‐dependent changes. In each hypermethylated CpG, DNAm levels tended to decrease with age, while in each hypomethylated CpG, DNAm levels tended to increase with age. All CpGs exhibited heteroscedasticity for the change in DNAm as a function of age, demonstrating that the drift could be due to increases in methylomic variability with increasing age. This phenomenon—observed for many CpGs—is commonly described in the literature. It is thought to occur due to environmental and stochastic processes that lead to the failure of DNAm maintenance over the course of repeated mitosis.^39^

### 4.2 Relationships among APOE methylation, AD, and cognition

Neither cognition nor family history of AD were associated with *APOE* methylation. This might be due to a lack of appreciable changes in *APOE* methylation before the onset of symptoms of AD. Most previous studies that associated differential *APOE* methylation with AD were conducted on tissue from patients that had either been screened positive for cognitive dysfunction,^15^ or diagnosed with AD^14^, ^16^, ^17^; only one study^19^ evaluated the relationship between DNAm and cognition in healthy participants. The epigenetic changes in *APOE* accompanying AD‐related cognitive decline could result from the pathophysiology of the disorder or from adaptive responses of the organism as a result of AD, neither of which may be present at appreciable levels in the population studied here. Another consideration concerns the tissue used. Foraker et al.^14^ used *post mortem* brain tissue, Karlsson et al.^15^ and Liu et al.^19^ used blood, while Shao et al.^16^ and Wang et al.^17^ used both. In fact, the latter were not able to replicate their findings from brain tissue in blood. Blood represents an attractive medium for identifying biomarkers for disease. Indeed, it has been reported that patients with AD and healthy controls can be distinguished based on gene‐expression patterns in blood.^40^ However, the *APOE* gene is differently expressed between brain tissue and blood^16^, ^17^ and *APOE* CpGs exhibit relatively modest correlations between blood and brain DNAm.^34^, ^35^, ^36^ Thus, blood‐based DNAm may exhibit AD‐associated changes that are distinct from DNAm changes in the brain or they might appear later in the course of the disorder. Finally, while some studies have reported AD‐associated changes in *APOE* methylation as described above, little research has been done on the topic and few—if any—replication studies have been performed to validate the effects. Moreover, some prominent studies that investigated associations between DNAm and AD across the entire genome did not report *APOE* to be altered in the disorder.^13^, ^41^

### 4.3 Relationship between APOE methylation and blood cholesterol

We did not replicate the finding^24^ of an association between *APOE* methylation and CVD; our results are in line with reports by Karlsson et al.^15^ and Sharma et al.^42^ However, we did find a negative association between *APOE* methylation and the ratio of the total to HDL cholesterol at cg08955609 and cg18768621; a finding that—to our knowledge—had not been reported before. However, due to the relatively small effect size, replication in other large cohorts or meta‐analyses is required to confirm these findings. Moreover, our measurements of cholesterol provide only levels of total blood cholesterol and of HDL cholesterol. Because of this—especially considering the opposite correlation of *APOE* genotype with the concentrations of LDL and VLDL, respectively^43^—it is not possible to assess the relationship between DNAm and the individual components of blood cholesterol.

### 4.4 Limitations and future directions

The results of the present study offer additional insight into the epigenetics of the *APOE* gene and its association with relevant phenotypes. The main strength of the study is the large sample size from a relatively representative sample of the Scottish population. Nevertheless, we recognize several limitations. First, the measures of risk for AD and CVD were inferred from information on participants’ relatives. The rough approximation of risk underestimates the importance of environmental factors. Moreover, different participants in our cohort have different numbers of relatives. Second, the study is limited to (a subset of) the methylome; other important epigenetic mechanisms that are currently less accessible to analysis at scale might also play important roles in the studied interactions. Third, our study is based on DNAm data from the blood, which does not necessarily correspond to DNAm patterns in the brain. This might preclude the identification of potential epigenetic changes prior to disease onset and allows only limited insight into underlying biological processes. However, due to the multitude of peripheral biological processes associated with AD, blood may be a legitimate tissue for DNAm studies. Finally, this study adopts a candidate gene approach; while *APOE* is a biologically plausible candidate for implications in AD and CVD, genome‐wide approaches may inform on the relative importance of *APOE*.

In conclusion, we showed that CpGs in the *APOE* gene exhibit correlations within and between distinct regions of the gene and that DNAm levels at some CpGs of the *APOE* gene correlate with the *APOE* genotype. Furthermore, we found an association between DNAm level at cg8955609 and cg18768621, and blood cholesterol. We did not find differences in the levels of *APOE* methylation between individuals at low risk and individuals at high risk of developing either AD or CVD. Future work might explore longitudinal changes in DNAm as it relates to adverse health outcomes to circumvent the problems of the present study and improve our understanding of the exact timing of epigenetic changes in AD and CVD.

## CONFLICTS OF INTEREST

Andrew M. McIntosh has received research support from Eli Lilly and The Sackler Trust. He has also received speaker fees from Illumina and Janssen.

## Supporting information

Supplementary information
